# The Insanity Law of the State of New York

**Published:** 1910-02

**Authors:** 


**The Insanity Law of the State of New York.** A compilation of statutes relating to the insane and to institutions for their care and treatment; to which are appended the official orders and regulations of the State Commission in Lunacy. By **Frank P. Hoffman,** Albany, N. Y.

This is a compilation of the insanity laws of this state, together with the rules, regulations, forms, and orders, salary, schedules, etc., prescribed by the department, and other matters of general interest as to the care and treatment of the insane in this state. The insanity laws were consolidated last year, and this publication contains all the changes made at the last session of the legislature. The price of the book is $1.00 and it is worth it.

